# Self-collected versus clinician-collected cervical samples for the detection of HPV infections by 14-type DNA and 7-type mRNA tests

**DOI:** 10.1186/s12879-021-06189-2

**Published:** 2021-05-31

**Authors:** C. E. Aranda Flores, G. Gomez Gutierrez, J. M. Ortiz Leon, D. Cruz Rodriguez, S. W. Sørbye

**Affiliations:** 1 Oncology Department, Hospital General de México “Eduardo Liceaga”, Mexico City, Mexico; 2 Department of Colposcopy, Hospital General de Mexico “Eduardo Liceaga”, Mexico City, Mexico; 3 Oncoly-Ginecology Department (R5), Hospital General de Mexico “Eduardo Liceaga”, Mexico City, Mexico; 4 Department of Clinical Pathology, University Hospital of North Norway, Tromsø, Norway

**Keywords:** Cervical cancer, Cervicovaginal self-sampling, Hr-HPV DNA, Cervical tumor markers, E6/E7 mRNA, XytoTest

## Abstract

**Background:**

HPV self-sampling has been widely supported by the scientific community following a strong body of literature on the subject. Self-sampling is important in cervical cancer screening as it has been shown to improve participation. It is well documented that HPV-testing has proven superior to cytology with regards to sensitivity in detection of CIN and cancer. The value of self-collected samples is reliant on the quality of the molecular testing performed, as well as the patients’ preference in sampling procedure and compliance to follow up on positive test results. Due to the incompatibility of self-samples and cytology, triage of HPV-DNA positives by testing for molecular biomarkers is highly warranted.

**Methods:**

Our objective was to compare the detection rate of genital Human Papillomavirus (HPV) infection in self- and clinician-collected samples by a 14-type HPV-DNA test and a 7-type mRNA E6/E7 test.

**Results:**

Five hundred five women were recruited. Each study participant had two sample collection procedures performed upon the same visit, alternating order in execution of the self-collection or the clinician-taken procedure first or second, 1010 samples in total. HPV-DNA prevalence was 22.8% in self-collected versus 19.2% in clinician-collected samples (*P* = 0.19). Overexpression of mRNA E6/E7 from 7 HPV types was 7.1 and 6.3%, respectively (*P* = 0.71). The difference between HPV-DNA and HPV-mRNA positivity rates were statistically significant in both self-collected (22.8% versus 7.1%, *P* < 0.001) and clinician-collected samples (19.2% versus 6.3%, *P* < 0.001). Overall agreement between the two collection methods was fair, with a concordance rate of 78.2% (390/505), k = 0.34 (95% CI: 0.25–0.44), *P* < 0.001, for the HPV-DNA test and 92.5% (467/505), k = 0.40 (95% CI, 0.25–0.56), *P* < 0.001, for the mRNA test, respectively. 96.8% of the participants reported they felt confident carrying out the self-collection themselves, and 88.8% reported no discomfort at all performing the procedure.

**Conclusions:**

This comparative study of two sampling methods reports fair agreement of HPV positivity rates between the self-collected and clinician-collected specimens using Abbott hrHPV and PreTect HPV-Proofer’7 tests. Only one third of HPV-DNA positive women had overexpression of mRNA E6/E7.

**Trial registration:**

ISRCTN77337300.

**Supplementary Information:**

The online version contains supplementary material available at 10.1186/s12879-021-06189-2.

## Background

Cervical cancer is a major public health problem, and the second most common cancer in women living in less developed regions with an estimated 570,000 new cases in 2018. According to the World Health Organization, approximately 311,000 women died from cervical cancer in 2018: more than 85% of these deaths occurring in low- and middle-income countries [1]. In Mexico, it is the third most common malignancy among women [2]. Infection by high-risk Human Papillomavirus (hr-HPV) of the cervicovaginal tract is known to be the major cause of cervical cancer [3]. HPV-detection in clinician-collected cervical samples has proven its superiority to cervical cytology in primary screening for prevention of cervical cancer [4, 5] and we are facing a paradigm shift towards molecular HPV-testing on a global perspective. Different national guidelines and follow-up algorithms are suggested, to maximize screening benefits. The shift will increase program sensitivity, however accurate triage of test positives is needed since Human Papillomavirus infection is the most common sexually transmitted viral disease in adult women. It is estimated that the vast majority of sexually active women will be exposed to the virus at some point in their lives [6]. Every programs success relies on a high coverage rate among the target population. Self-collection of cervical samples is reported to be highly acceptable and preferred by most women, being a promising approach to enhance women’s participation in regular screening for cervical cancer prevention [7]. It offers significant benefits over conventional sampling in terms of cost, coverage and convenience for patients. Self-sampling reaches high-risk groups who currently have limited access to national health system screening for personal and practical reasons [8]. Numerous studies comparing self-collected and clinician-collected samples for HPV detection show good concordance when clinically validated PCR-based methods are used [9, 10]. In a meta-analysis by Arbyn et al., self-sampled HPV tests based on PCR for the detection of CIN2+ were shown not to have statistically different sensitivity or specificity compared with clinician-sampled tests [11].

Currently, most guidelines recommend cytology in triage of HPV-DNA positives. As PAP reflex testing is not applicable on self-collected material, a new clinician-collected sample is required for the purpose of triage, hereby suffering increased risk of loss to follow up; based on the nature of non-attenders. An alternative to cytology in triage could be detection of molecular biomarkers; being compatible on self-sampled material [12].

Literature describes effects caused by device and specimen processing in terms of various concentration and quality of cellular material that possibly impair test performance [11, 13] while few studies have evaluated the feasibility to collect sufficient material for both primary HPV-testing with direct molecular triage.

This study was conducted to compare the performance of self-collected samples by using a self-sampling device (XytoTest, Mel-Mont Medical, US) versus clinician-collected samples by using the professional-use device (Cervex-Brush, Rovers Medical Devices, Oss, the Netherlands) in combination with a 14-type HPV-DNA and 7-type mRNA E6/E7 test as the primary outcome.

Patient-reported acceptability of the self-collected samples method was the only secondary outcome.

## Methods

The study was approved by the institutional ethics review board (CI/243/18) at Eduardo Liceaga, Mexico General Hospital, Mexico City, before commencing.

Trial registration: ISRCTN, ISRCTN77337300. Registered 15 December 2020 - Retrospectively registered, http://www.isrctn.com/ISRCTN77337300

During the period August 2018 to April 2019, study participants were recruited among women attending cervical cancer screening and among health professionals working at the Oncology clinic of Eduardo Liceaga, Mexico General Hospital, Mexico City. Eligible were sexually active women aged 30–65 that responded positively to the invitation and had no history of medical or surgical treatment (radiotherapy, chemotherapy, hysterectomy, cone biopsy) for cervical cancer. Excluded were pregnant and breastfeeding women, those who had had sexual activity within 24 h before to the collecting samples procedure, and those who chose not to sign the informed consent. Prior to enrollment, the purpose and nature of the study were explained to each participant to obtain their written consent. The two sampling procedures were performed upon the same day, in alternating order for each participant upon arrival. Since the study was conducted at the clinic for cervical screening, clinicians’ additional training was unnecessary to collect the reference sample. For the self-sampling device, the manufacturer’s written and illustrated instructions for use in Spanish were provided to all participants. The procedure was performed in a separate room for the women’s discretion without assistance by health staff. Finally, after the samples were collected, the participant completed a questionnaire about her experience during the self-collection procedure. Respondents scored items on an 8-point Likert scale, ranging from 1 (“no discomfort”) to 8 (unbearable discomfort”). All women were informed to discuss their test results upon completion with a healthcare professional to decide on further follow-up. Patient sensitive information was anonymized by a 6-digit code and registered using Microsoft Excel 16.16.23 (200615–2016). Only study responsible staff at the hospital had access to the registered information.

### Self-collected samples

The participants of this study performed self-collection of cervicovaginal material using the XytoTest medical device (Mel-Mont Medical, USA), an ergonomically designed device to be inserted into the vagina, with a diameter less than 8 mm and a length of 14 cm [14]. The cell collection area is made of USP medical grade IV elastomer, allowing for immediate collection of cells when inserted into the vaginal canal. A chemical release of cells occurs when resuspending the device in a methanol-based preservative [14]. The instruction leaflet (attached as supplementary file; S1) guides women to collect the sample from a gynecological position; laying on their back with their legs bent, spread the labia with one hand and carefully insert the device into the vagina with the other until the lower flap of the device contacts the skin and slowly rotate it 360° in the same direction three times before retracting the device and placing it in the container provided. The sample was given to the clinician, who thoroughly washed the device in 5 ml of PreservCyt Solution (Hologic, UK) for at least 2 min to release cell material for subsequent HPV-testing.

### Clinician-collected samples

A clinician collected the sample by using the Cervex-Brush® (Rovers Medical Devices, Oss, the Netherlands), which is among the most widely used method for cervical cancer screening, commonly used as a reference method for validation of new devices [15]. After sampling according to the manufacturer’s instructions, the brush was immediately rinsed in 5 ml of PreservCyt Solution (Hologic, UK) for subsequent HPV-testing. Cellularity was calculated for 19.2% (97/505) of the paired samples collected by performing a manual cell count using the Bürker Chamber [16].

### HPV-DNA test

The Abbott RealTi*m*e HR HPV test (Abbott, Wiesbaden, Germany) is an automated, qualitative multiplex assay based on real-time polymerase chain reaction (PCR) intended to detect 14 high-risk HPV genotypes (16, 18, 31, 33, 35, 39, 45, 51, 52, 56, 58, 59, 66, 68) and to partially genotype 16, 18 from the other 12 high risk genotypes. Human Beta-globin is detected as an internal control. The test has been clinically validated according to international consensus guidelines and proved to also be accurate for self-collected samples [17, 18]. All samples were tested using the standard procedure and interpreted according to the manufacturer’s threshold for positivity CT < 32.0. Any invalid sample with respect to Beta-globin was retested and excluded from the study population if repeatedly invalid.

### HPV mRNA E6/E7 test

PreTect HPV-Proofer’7 (PreTect AS, Klokkarstua, Norway) is a diagnostic kit for the qualitative detection and direct typing of E6/E7 mRNA from HPV 16, 18, 31, 33, 45, 52 and, 58. The kit contains an intrinsic sample control (ISC) targeting a human housekeeping gene to assess specimen quality and reveal possible factors that may inhibit amplification. The kit utilizes real-time NASBA technology, an enzymatic one-step amplification process able to amplify RNA under isothermal conditions at 41 °C [19]. Several publications describe PreTect HPV mRNA assays’ clinical performance, holding high specificity in low-grade cytology triage [19–21]. All samples were tested and interpreted according to the manufacturer’s standard procedure. Any invalid sample with negative mRNA intrinsic sample control was retested and excluded from the study population if repeatedly invalid.

### Statistical analysis

The sample size was estimated considering a two-tailed hypothesis, with a 95% confidence interval, a statistical power of 80%, and an effect size (Cohen’s d) of 0.12555. With these characteristics, the estimated sample size required was 500 study participants, calculated using the software GPower 3.1.9.2. Microsoft Excel 2016 (Microsoft Corp., Redmond, WA) and IBM SPSS Statistics software package version 21 (IBM, Armonk, NY, USA) were used for data collection and evaluation. Cohen’s Kappa coefficients were calculated to evaluate the agreement between the two sampling methods regarding hr-HPV results, applying the most commonly used scale to express the strength of the agreement as follows: 0.00–0.20, 0.21–0.40, 0.41–0.60, 0.61–0.80 and ≥ 0.81 indicated slight, fair, moderate, substantial, and almost perfect, respectively. The Wilcoxon signed -rank test was applied to evaluate the differences between the two sampling methods. A statistically significant difference was defined as a 5% chance of a type I error (α ≤0.05). For comparison of the presence of HPV-16, HPV-18, and 12 other hr-HPV genotypes between the two samples, the following terminology was used: concordant or discordant. A concordant result was determined if results from all three channels (16, 18, and pool) showed at least one identical genotype in both samples, where discordance represented no similarities in genotypes at all.

## Results

### Participant characteristics

Overall, a total of 505 women participated in the study, everyone within the inclusion criteria. 65.5% of the participants were recruited among women attending the general screening program at the clinic, and 33.5% were recruited among the health professionals working at the Mexico General Hospital. The inclusion of health professionals was partially to learn whether the level of education might affect the quality of the sample evaluated by cellularity and sample integrity if the instructions for self-collection was not sufficiently clearly described as health professionals presumably would have a better understanding of sample taking in general. In total 1010 samples were collected equally distributed between self-collected samples, and clinician collected specimens. All the collected samples by the two procedures were valid for processing. The mean age of the participants was 43.8 ± 8.1 years (median 44, IQR 13, range; 30–63). Just over two-thirds of the women (69.7%) reported they were older than 18 years when they first had sexual intercourse, and less than a third (30.3%) were under the age of 18 (Table 1).

**Table 1:** Characteristics of study population, *n* = 505

Age (years)		
Mean ± SD	43.8	±8.1
Median ± IQR	44	±13
Recruitment source	n	(%)
Health professionals	169	(33.5)
Women attending screening	336	(66.5)
Age at first sexual intercourse		
< 18 years	153	(30.3)
> 18 years	352	(69.7)

### Questionnaire

Each of the four questions’ response rate in the questionnaire was high, ranging between 99.0–99.2%. Regarding women’s acceptability of the self-sampling procedure, 88.8% reported no discomfort at all, 94.0% found no difficulty performing the self-sampling procedure, 96.6% agreed they would perform self-sampling again, and 96.8% said they felt confident carrying out the procedure themselves (Table 2).

**Table 2:** Questionnaire Responses (acceptability of self-collection, *n* = 505)

	(n)^a^	(%)
**Level of discomfort**		
1	445	88.8
2	32	6.4
3	10	2.0
4	7	1.4
5	1	0.2
6	3	0.6
7	2	0.4
8	1	0.2
(Total responses)	501	99.2
**Level of difficulty**		
1	471	94.0
2	21	4.2
3	4	0.8
4	1	0.2
5	1	0.2
6	1	0.2
7	1	0.2
8	1	0.2
(Total responses)	501	99.2
**Would you perform self-sampling again?**		
Yes	483	96.6
No	17	3.4
(Total responses)	500	99.0
**Do you feel confident taking the sample?**		
Yes	484	96.8
No	16	3.2
(Total responses)	500	99.0

^a^*n* number of responses obtained

### Hr-HPV prevalence

The prevalence of hr-HPV-DNA varied from 22.8% (115/505; 95% CI: 19.2–26.7) to 19.2% (97/505; 95% CI: 15.9–22.9) among the self-collected and clinician-collected samples (*P* = 0.19), whilst the positivity rate for HPV mRNA E6/E7 was about one third; 7.1% (36/505; 95% CI: 5.0–9.7) versus 6.3%, (32/505; 95% CI: 4.4–8.8) respectively (*P* = 0.71).

The Wilcoxon test showed no statistically significant differences between the two sample-collection methods used prior to analysis by DNA and mRNA assays (*P* > 0.05). Mostly non-HPV16/18 genotypes were detected. The vast majority of the infections identified in self-collected samples were single (85.2%) and multiple infections were found in 17 specimens (14.8%) in line with 84.5 and 15.5% among clinician-collected samples (Table 3). The analysis of HPV genotype infections was restricted to count each infection only once and results for both HPV-DNA and mRNA are shown hierarchically based on the established oncogenicity for cervical cancer. For self-collected samples tested by Abbott hr-HPV; a total of 16 out of 505 samples (3.2%) showed single or multiple infections with genotype HPV-16; 8 samples (1.6%) showed single or multiple infections with HPV-18 excluding any co-infections with HPV-16. A further 91 samples (18.0%) showed single (or multiple) infections with non-16/18-HPV genotypes. For clinician-collected samples the results were 15/505 (3.0%), 6 (1.2%) and 76 (15.0%), respectively (Table 3). To have comparable statistics, the HPV mRNA genotype results were presented in the same way, HPV mRNA-16 (single and multiple), HPV mRNA-18 (non16), and HPV mRNA-31, 33, 45, 52, 58 positive (non16/18) samples (Table 3). Overall agreement between the two collection methods was fair, with a concordance rate at 78.2% (395/505), k = 0.34 (95% CI: 0.25–0.44), *P* < 0.001, for the HPV-DNA test and 92.5% (467/505), k = 0.40 (95% CI: 0.25–0.56), *P* < 0.001, for the HPV mRNA test, respectively. In women with at least one positive HPV-test, the agreement was 31.7% (51/161) for the HPV-DNA test and 28.3% (15/53) for the HPV mRNA test. Kappa for the mRNA test in the 161 women with at least one positive HPV DNA test was 0.30 (95% CI: 0.12–0.48), *P* < 0.001 (Table 4).

**Table 3:** Partial hr-HPV genotyping results presented hierarchically by oncogenicity for clinician-collected (CC) and self-collected (SC) samples

	14-type DNA test		7-type mRNA test	
	CC	SC	CC	SC
	n (%)	n (%)	n (%)	n (%)
**N = 505 included cases**				
HPV 16	15 (3.0)	16 (3.2)	7 (1.4)	9 (1.8)
HPV 18 (non 16)	6 (1.2)	8 (1.6)	4 (0.8)	5 (1.0)
HPV other (non 16/18)	76 (15.0)	91 (18.0)	21 (4.2)	22 (4.4)
Any hr-HPV	97 (19.2)	115 (22.8)	32 (6.3)	36 (7.1)
**N = Any positive hr-HPV**				
Any multiple infections	15 (15.5)	17 (14.8)	3 (9.4)	7 (19.4)
Any single infections	82 (84.5)	98 (85.2)	29 (90.6)	29 (80.6)

**Table 4:** Agreement of self- and clinician-collected samples by HPV assay

		Clinician-collected samples						
		HPV positive	HPV negative	Total	Cohens kappa		Agreement	
	Self-collected	n (%)	n (%)	n (%)	κb	(95% CI)	%	(95% CI)
**14-type**	HPV positive^a^	51 (10.1)	64 (12.7)	115 (22.8)				
**DNA**	HPV negative	46 (9.1)	344 (68.1)	390 (77.2)			78.2^c^	(74.6–81.8)
**assay**	Total	97 (19.2)	408 (80.8)	505 (100.0)	0.34^e^	(0.25–0.44)	31.7^d^	(24.7–39.5)
**7-type**	HPV positive^b^	15 (3.0)	21 (4.2)	36 (7.1)				
**mRNA**	HPV negative	17 (3.4)	452 (89.5)	469 (92.9)	0.40^e^	(0.25–0.56)	92.5^c^	(90.2–94.8)
**assay**	Total	32 (6.3)	473 (93.7)	505 (100.0)	0.30^f^	(0.12–0.48)	28.3^d^	(17.2–42.3)

^a^ (16, 18, 31, 33, 35, 39, 45, 51, 52, 56, 58, 59, 66, 68)

^b^ (16, 18, 31, 33, 45, 52, 58)

^c^ Agreement overall

^d^ Agreement in women with at least one positive HPV test

^e^ Cohen kappa overall (*N* = 505)

^f^ Cohen kappa in women with at least one positive HPV DNA test (*N* = 161)

A total of 115 women (22.8%) had discordant HPV-DNA results. In 46 cases, the women were clinician-collected sample HPV-positive/self-collected sample HPV-negative. Among these, 24 had the clinician-collected sample taken first and 22 women the self-sampling first in order. Sixty-four women were clinician-collected sample HPV-negative/self-collected sample HPV-positive; among those of whom 27 had their first sample by their clinician versus 37 women self-sampling first. Five women were detected as hr-HPV positive in both samples collected, with no similarities in genotypes. Regardless of order of the method used, no significant differences in the agreement rates for self-collected, and clinician-collected sampling techniques were observed, *P* = 0.63 for the HPV-DNA test and *P* = 0.23 for HPV mRNA test, respectively (Table 5).

**Table 5:** Paired agreement and order of procedure for Clinician-collected (CC) and Self-collected (SC) samples

	14-type DNA^a^			7-type mRNA^b^		
Paired Agreement	n	CC first	SC first	n	CC first	SC first
Concordant	390	195	195	466	225	241
Only Clinician-collected sample positive	46	24	22	17	10	7
Only Self-collected sample positive	64	27	37	21	14	7
Both samples positive, Different genotypes	5	3	2	1	0	1
Total	505	249	256	505	249	256

^a^ *p* = 0.63, Pearson’s Chi-squared test^b^ *p* = 0.23, Pearson’s Chi-squared test

### Cellularity

All 505 paired samples obtained valid results for the two subsequent HPV amplification tests done, hence no exclusions were made due to low cellularity. The manual cell count using Bürker chamber was done for a random subset of paired samples to evaluate the number of cells collected by each sampling method, 97 pairs in total. Forty-seven of the self-collected samples were done by health professionals, 50 self-collected by women attending screening. The average number of cells per milliliter was calculated following standard protocol and descriptive statistics are presented in (Table 6). The self-sampled aliquot contained about 3 times more cells compared to clinician taken aliquot; 1.87 million cells/ml versus 0.63 million cells/ml, respectively. The difference in cell counts between the two methods was significantly different from 0 (*P* < 0.001), tested using Wilcoxon signed rank test.

**Table 6:** Descriptive statistics for cellularity (number of cells/mL)

Sample	N	Min.	Max.	Mean	Median	1st Qu.	3rd Qu.
Clinician-collected	97	13,333	6,880,000	630,653	293,333	146,667	546,667
Self-collected^a^	97	13,333	10,866,667	1,866,804	1,600,000	773,333	2,733,333
-SC (health prof.)^b^	47	93,333	5,733,333	1,647,943	1,266,667	653,333	2,560,000
-SC (attn scr.)^b^	50	13,333	10,866,667	2,072,533	1,906,667	980,000	2,753,333
Valid N (listwise)	97						

^a^ Self-collected samples had significantly higher cellularity than Clinician-collected samples (*P* < 0.001)

^b^ Self-collected samples by health professionals had similar cellularity to self-collected samples by women attending screening (*P* = 0.19)

Evaluating the cellularity obtained by self-collection performed by the two subgroups of study participants (health professionals/women attending screening), no difference was observed (*P* = 0.19).

## Discussion

### Main findings

Our study showed fair concordance in HPV detection between the paired self-collected and clinician-collected samples, with no statistically significant differences between the two procedures. Both sampling methods provided material of sufficient quality and cellularity for molecular diagnostics. The self-collecting procedure was well accepted among study participants.

### Methodology and results

Overall, enabling women to perform self-collection of samples is an important avenue in facilitating greater coverage and participance in cervical cancer screening programs. Self-sampling is considered to improve the subjective patient experience, increase screening coverage, and ultimately reduce morbidity and mortality related to HPV infection and cervical cancer [22]. In Mexico, cervical cancer is still a major public health problem. An infrastructure is in place for national cervical cancer prevention and early detection programs in most Mexican states, but organization is inefficient and implementation is poor [23]. This was an essential motive for conducting this study among the Mexican population. With evidence to support the performance of the self-sampling method it can be possible to justify implementing this method in national screening programs, enabling women to receive and send samples remotely, without the need to visit a clinic which can be an obstacle to participation, thus increasing coverage and participation numbers to further decreasing figures for mortality and incidence of cervical cancer.

As in every difficult to reach population, there is a considerable risk of loss to follow-up as a consequence of inadequate self-collected samples or the need of a clinician-collected sample for triage purposes. Some studies reflect the importance of sample quality and number of samples to be rejected due to low cellularity. In this study, all paired samples had sufficient sample material allowing two individual molecular tests to be done, with no need for revisits due to invalid test results. Since both tests provide qualitative results with no accurate quantification of pathogen load, the influence caused by the observed higher cellularity per milliliter in self-collected sample aliquot for HPV-testing needs to be interpreted with caution. Cut-off values for HPV positivity established by manufacturer (CT < 32.0) is designed to optimize clinical performance, and since no relevant clinical outcome by the means of cytology or histology is available in this study, this will not be possible to discuss. It is well known that PAP test sensitivity [24] varies dramatically, highly impacted by sampling technique [25] emphasizing the importance of an optimal combination of sampling techniques to increase screening participation with a highly specific molecular triage strategy to manage the positive results following HPV testing.

Within this study population, the hr-HPV detection rate by PCR assay was about 20%, which is comparable to the prevalence (24.7%) found in another study conducted in the Mexican population [26]. Clinical relevance caused by detecting vaginal HPV is not possible to conclude in our study set-up, in the absence of corresponding histology results. However, previous research shows that HPV is present beyond the transformation zone and the consequence of HPV infection differs depending on the site of infection in the cervix [27]. Both sampling techniques were performed upon the same visit, with the likelihood to collect infected cells by the first sampling method resulting in insufficient cell material for next sample procedure to reproduce HPV positivity. However, our results showed that the order of sampling was found not to be statistically significant for any discordant HPV result.

The obtained significant higher cellularity per milliliter for the self-collected versus the clinician-collected specimens in this study might be explained by the fact that XytoTest has an area coated with a highly adhesive hypoallergenic elastomer and therefore cells are more readily collected as soon as the device is inserted into the vagina.

### Perspectives

It is known that molecular biomarkers are of vital importance in relation to the triage of patients in cervical screening programmes [28]. All current screening programs that utilize HPV-testing rely on cytology for triaging positive samples prior to referral for colposcopy. However, cytology is subjective in nature and the widespread implementation of this practice leads to over-referral, particularly for low-grade cellular abnormalities, which continues to be challenging from the clinical perspective [29].

The perspective of implementing HPV mRNA in triage of test positives are among one of the options discussed to represent an improved, highly specific risk-based approach for maximizing screening benefits and minimizing harms [19–21, 30].

Our data confirm 7-type mRNA testing to be applicable for reflex testing on self-collected specimens. Among the study participants, only one third of the HPV infected women had a positive mRNA test, an appealing situation for effective triage reducing the number of colposcopies and biopsies.

Recent research has proven the 7 genotypes included in the mRNA test to be the most important to screen for, being responsible for 90% of all cervical cancers [29, 31, 32].

Reliable self-sampling methods and molecular diagnostics may significantly aid prevention of cervical cancer, by simplicity, increased accessibility to screening and accurate diagnostics.

By combining HPV-DNA testing and identification of mRNA E6/E7 biomarkers, both high sensitivity and specificity might be maintained for improved patient management [33, 34].

### Limitations

A clear limitation of this study is the lack of clinical data; cytology and a histology examination of samples that were found to be hr-HPV positive or mRNA E6/E7 positive, which would have enabled associations to be drawn between molecular assays with possible morphological changes, and presumably identify progression of cervical lesions. Unfortunately, these data were not available for our review, nor contemplated in the inclusion criteria of this study.

Although the acceptability of self-collected samples was found to be high among the women participating in this study, the questionnaires are of limited reliability because the study administered no parallel healthcare provider-reported questionnaire.

## Conclusions

This comparative study of two sampling methods reports fair agreement of hr-HPV positivity rates between the self-collected and clinician-collected specimens using Abbott hr-HPV and PreTect HPV-Proofer’7 tests. Only one third of HPV-DNA positive women had overexpression of mRNA E6/E7, effectively discriminating women warranted for immediate colposcopy/biopsy from return to follow-up and suggests longer follow-up interval for single HPV-DNA positive women. Such a strategy will inevitably reduce over-referral for colposcopy but needs clinical and cost-benefit assessment in prospective studies.

## Supplementary Information

**Additional file 1.**

## Data Availability

The dataset used in this study contains personal information and is not publicly available, however an anonymized dataset is available from the corresponding author on reasonable request.
