# Supplementary material for: Self-collected versus clinician-collected cervical samples for the detection of HPV infections by 14-type DNA and 7-type mRNA tests
Source: BMC Infect Dis. 2021 May 31;21:504. doi: 10.1186/s12879-021-06189-2 (PMC8165795; doi:10.1186/s12879-021-06189-2)
Supplement: Supplementary file 1 — Additional file 1. [file 12879_2021_6189_MOESM1_ESM.pdf]

How to use
Mía by **XytoTest®**

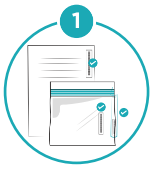

Verify that the barcode on the test tube matches both the barcode on the Consent Form and the barcode on the security envelope where the sample will be transported.

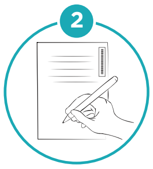

Complete all the fields of the informed consent.

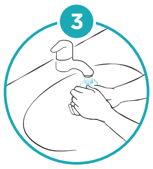

Wash your hands and / or put on gloves before using the device **Mía** by **XytoTest®**.

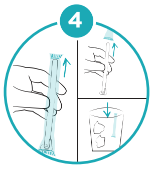

Take out the device **Mía** by **XytoTest®** opening and discarding the cellophane packaging that covers it. **Remove the protective cap from the device and throw it away.**

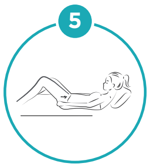

The person who is going to take the sample must be in a gynecological position, that is, lying on their back and with their legs bent.

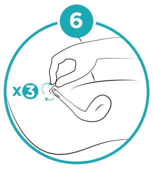

Spread the labia with one hand and carefully insert the device into the vaginal canal with the other until the lower flap of the device makes contact with the skin. Once the device is inserted, rotate it **three times** (360 °) in the same direction.

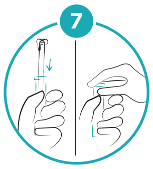

Carefully remove the device from the vaginal canal and immediately insert into the cylinder included in the **Xytotest®** kit.

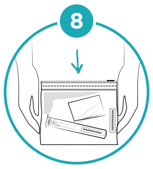

Make sure to close the tube correctly and insert it together with the Consent Form in the security bag. Deliver the sample as soon as possible for analysis according to the logistics system indicated by the laboratory.

MEL-MONT MEDICAL

Mía by XytoTest
Prevention in your own hands
